# What Kafka’s *Metamorphosis* reveals about consciousness without communication

**DOI:** 10.1093/braincomms/fcag331

**Published:** 2026-09-11

**Authors:** Alexis Demas

**Affiliations:** ARGOS (Arts, Research and Gestures Observed Scientifically) Neurosciences and Arts, Paris 75001, France

***Gregor Samsa awakens transformed. His body has become unrecognizable, his movements incoherent and his voice unintelligible. Yet nothing in his inner life appears altered. He thinks clearly, remembers precisely and understands his situation with painful lucidity. From the opening lines of The Metamorphosis, consciousness is intact, but its expression has collapsed.***

This dissociation is not merely literary. It mirrors a fundamental challenge in modern neurology: the possibility that awareness persists in the absence of communication. In conditions such as locked-in syndrome, patients may remain fully conscious while losing almost all voluntary motor output. They are awake, attentive and cognitively preserved, yet unable to speak or gesture. The problem is not consciousness itself, but access to it.

Kafka’s narrative captures this gap with unsettling precision. Gregor attempts to communicate, but his speech becomes incomprehensible, and his movements are misread as erratic or threatening. His intentions persist, but their interpretation fails. What is lost is not thought, but translation. The tragedy lies in this asymmetry: a mind that continues, and a world that can no longer recognize it.

In clinical practice, consciousness is never directly observed. It is inferred from behaviour. When behaviour is profoundly altered, this inference becomes fragile. Studies have shown that disorders of consciousness are frequently misdiagnosed, with patients sometimes considered unaware despite evidence of preserved cognitive processing.^1^ In such contexts, the absence of communication risks being mistaken for the absence of awareness.

Kafka anticipates not only this diagnostic problem but also its ethical consequences. As Gregor loses the ability to make himself understood, his status within the family shifts. He is no longer treated as a subject with intentions, but as an object to be managed. Decisions about his care are made without access to his perspective. His existence becomes progressively reduced to its external manifestations.

This dynamic echoes real-world dilemmas in the care of patients with severe neurological impairment. Decisions regarding life-sustaining treatment often rely on assumptions about the patient’s level of consciousness and quality of life. When communication is absent, these assumptions may be uncertain. The risk is not only a diagnostic error but also an ethical misjudgement.

At the same time, modern medicine has begun to challenge these limits. Functional neuroimaging has demonstrated that some patients diagnosed as being in vegetative or minimally conscious states retain covert awareness, detectable through patterns of brain activation during specific cognitive tasks.^2^ These findings suggest that the boundary between consciousness and its absence is less clear than previously assumed. They also reinforce the need for diagnostic humility.

Efforts to restore communication further complicate this landscape. Even minimal voluntary movements, such as eye movements, can provide a channel for interaction. More recently, brain–computer interfaces have begun to offer alternative pathways, translating neural activity into communicative output.^3^ These technologies do not create consciousness; they reveal it.

Yet, Kafka’s insight extends beyond the patient. The metamorphosis does not affect Gregor alone. It reorganizes the entire family. His sister initially responds with care and attention, attempting to interpret his needs. Over time, this attentiveness erodes, replaced by fatigue and withdrawal. The father reasserts authority through aggression, culminating in acts of violence that contribute to Gregor’s death. What begins as a biological transformation becomes a relational one.

In this sense, The Metamorphosis describes a system under strain. The patient’s inability to communicate transforms not only his own condition, but the behaviour, identity and moral positioning of those around him. Illness becomes a force that reshapes relationships.

The final lines of the narrative extend this logic further. After Gregor’s death, the family experiences a form of release. The sister, once burdened by caregiving, is described as stretching and ‘unfolding’ her body, an image that echoes the language of metamorphosis itself. Transformation persists, but its direction has reversed. What was once deformation becomes expansion.

This closing image reframes the entire story. The metamorphosis is not confined to Gregor’s body; it circulates through the family, redistributing roles, emotions and identities. In clinical terms, the patient’s condition cannot be isolated from its relational context. The absence of communication does not produce silence; it produces reinterpretation.

Kafka’s text therefore offers more than a metaphor for neurological disease. It provides a conceptual framework for understanding the limits of observable behaviour, the risks of misinterpreting consciousness and the ethical consequences of uncertainty. It reminds clinicians that behind the absence of communication, there may still be a mind that perceives, understands and experiences.

The challenge is not only to detect consciousness but also to resist the impulse to equate silence with its absence.
